# International Society of Sports Nutrition position stand: energy drinks

**DOI:** 10.1186/1550-2783-10-1

**Published:** 2013-01-03

**Authors:** Bill Campbell, Colin Wilborn, Paul La Bounty, Lem Taylor, Mike T Nelson, Mike Greenwood, Tim N Ziegenfuss, Hector L Lopez, Jay R Hoffman, Jeffrey R Stout, Stephen Schmitz, Rick Collins, Doug S Kalman, Jose Antonio, Richard B Kreider

**Affiliations:** 1 Exercise and Performance Nutrition Laboratory, Dept. of Physical Education and Exercise Science, University of South Florida, 4202 E. Fowler Avenue, PED 214, Tampa, FL, 33620, USA; 2 Human Performance Laboratory, University of Mary Hardin-Baylor, Belton, TX, 76513, USA; 3 Department of Health, Human Performance, and Recreation, Baylor University, Box 97313, Waco, TX, 76798, USA; 4 Department of Health and Human Performance, University of St.Thomas, St. Paul, MN, 55105, USA; 5 Exercise & Sport Nutrition Lab, Department of Health & Kinesiology, Texas A&M University, College Station, Texas, TX, 77843-4243, USA; 6 The Center for Applied Health Sciences, Stow, OH, 44224, USA; 7 Institute of Exercise Physiology and Wellness, Department of Sport and Exercise Science, University of Central Florida, Orlando, FL, 32816, USA; 8 Medical Surveillance and Risk Management, Shire HGT, 300 Shire Way, Lexington, MA, 02421, USA; 9 Collins, McDonald & Gann, PC, Mineola, NY, USA; 10 Miami Research Associates, Endocrinology & Nutrition Department, 6141 Sunset Drive - Suite 301, Miami, FL, 33143, USA; 11 Farquhar College of Arts and Sciences, Nova Southeastern University, Fort Lauderdale, FL, USA

## Abstract

Position Statement: The International Society of Sports Nutrition (ISSN) bases the following position stand on a critical analysis of the literature on the safety and efficacy of the use of energy drinks (ED) or energy shots (ES). The ISSN has concluded the following. 1. Although ED and ES contain a number of nutrients that are purported to affect mental and/or physical performance, the primary ergogenic nutrients in most ED and ES appear to be carbohydrate and/or caffeine. 2. The ergogenic value of caffeine on mental and physical performance has been well-established but the potential additive benefits of other nutrients contained in ED and ES remains to be determined. 3. Consuming ED 10-60 minutes before exercise can improve mental focus, alertness, anaerobic performance, and/or endurance performance. 4. Many ED and ES contain numerous ingredients; these products in particular merit further study to demonstrate their safety and potential effects on physical and mental performance. 5. There is some limited evidence that consumption of low-calorie ED during training and/or weight loss trials may provide ergogenic benefit and/or promote a small amount of additional fat loss. However, ingestion of higher calorie ED may promote weight gain if the energy intake from consumption of ED is not carefully considered as part of the total daily energy intake. 6. Athletes should consider the impact of ingesting high glycemic load carbohydrates on metabolic health, blood glucose and insulin levels, as well as the effects of caffeine and other stimulants on motor skill performance. 7. Children and adolescents should only consider use of ED or ES with parental approval after consideration of the amount of carbohydrate, caffeine, and other nutrients contained in the ED or ES and a thorough understanding of the potential side effects. 8. Indiscriminant use of ED or ES, especially if more than one serving per day is consumed, may lead to adverse events and harmful side effects. 9. Diabetics and individuals with pre-existing cardiovascular, metabolic, hepatorenal, and neurologic disease who are taking medications that may be affected by high glycemic load foods, caffeine, and/or other stimulants should avoid use of ED and/or ES unless approved by their physician.

## Introduction

According to published research, energy drinks (ED) are the most popular dietary supplement besides multivitamins in the American adolescent and young adult population [1-3]. ED are also reported to be the most popular supplement among British athletes [4]. More recently, energy shots (ES) have also been purported to possess ergogenic value on mental focus and/or performance [5]. It is important to make a distinction between ED, ES, and sports drinks. Sports drinks are a unique category within the beverage industry and are marketed to consumers with the primary function of promoting hydration, replacing electrolytes and sustaining endurance performance capacity. They typically provide a small amount of carbohydrate (e.g., 6-8 grams/100 ml) and electrolytes (sodium, potassium, calcium, magnesium). ED, on the other hand, typically contain higher amounts of carbohydrate along with nutrients purported to improve perceptions of attention and/or mental alertness. Low calorie ED are also marketed to increase mental alertness, energy metabolism, and performance. Energy shots are typically 2-4 oz. servings of concentrated fluid containing various purported ergogens. Since ED and ES contain carbohydrate, caffeine, and/or nutrients that may affect mental focus and concentration, they have the potential to affect exercise capacity and perceptions of energy and/or fatigue. The purpose of this position stand is to critically evaluate the scientific literature and make recommendations in regards to the role that ED and/or ES may have on exercise performance and energy expenditure/metabolism. Additionally, we will discuss safety considerations in regards to the use of ED and/or ES.

## Methods

This analysis represents a systematic review of the literature on the effects of “energy drinks” on exercise and cognitive performance as well as primary ingredients contained in popular energy drinks. A comprehensive literature search was performed by searching the Medline database of the US National Library of Medicine of the National Institutes of Health. The search strategy involved entering “energy drinks” and commercial names of energy drinks and/or caffeinated beverages as well as a search of primary nutrients contained in popular energy drinks (e.g., caffeine, carbohydrate, taurine, glucoronolactone, Guarana, Yerba Mate, etc.). It is important to note, from a United States regulatory perspective, several of these ED are marketed as dietary supplements and not beverages, and the label on the product will indicate which category of Food and Drug Administration (FDA) authority the product falls under. Each category has its own set of governing laws and regulations. For example, depending on the category, the labels will include Supplement Facts (dietary supplements) or Nutrition Facts (beverages). A paper summarizing the literature related to ED was presented at the 2011 International Society of Sports Nutrition Annual meeting. Thereafter, a position stand writing team was organized to develop this paper. Drafts of this position stand were then reviewed by all authors as well as the Research Committee of the International Society of Sports Nutrition (ISSN). The final version of this paper was then adopted as the official position of the ISSN.

## Ergogenic/performance considerations

The ingestion of nutrients prior to, during, and/or following exercise can affect exercise performance and/or training adaptations [6]. ED typically contain water, carbohydrates (e.g., glucose, maltodextrin), vitamins, minerals, and “proprietary blends” of various nutrients purported to increase energy, alertness, metabolism, and/or performance (e.g., caffeine, taurine, amino acids, glucoronolactone, Guarana, Ginkgo biloba, Carnitine, Panax ginseng, Green Tea, Yerba Mate, etc.). Therefore, ingestion of ED or ES prior to, during, and/or following exercise could have some ergogenic value. Tables 1 and 2 present a list of ingredients found in several ED/ES marketed in the United States. The next section provides an overview of the potential ergogenic value of some of the most commonly found nutrients in ED/ES.

**Table 1:** List of ingredients described on nutrient panels found in some energy drinks

**Ingredient**	**A**	**B**	**C**	**D**	**E**
Calories	100	110	140	120	140
Carbohydrate	27	28	31	32	30
Calories from Fat	-	-	-	-	
Vitamin C	-	100 mg	-	100 mg	6.7mg
Thiamin	-	0.1 mg	-	-	0.2 mg
Riboflavin B2	1.7 mg	1.5 mg	3.4 mg	20%	0.2 mg
Niacin B3	20 mg	21.7 mg	20 mg	10%	2.2 mg
Vitamin B6	40 mg	2.1 mg	2 mg	10%	1.5 mg
Vitamin B12	6 mcg	4.5 mcg	6 mcg	10%	4.7 mcg
Pantothenic Acid	-	36 mg	-	10%	1.1 mg
Sodium	180 mg	200 mg	40 mg	75 mg	190 mg
Potassium	-	10 mg	10 mg	-	65 mg
Phosphorus	-	-	-	40 mg	90 mg
Taurine	1,000 mg	600 mg	1000 mg	-	-
Panax Ginseng	200 mg	-	25	-	-
Proprietary Blend	2,500 mg L-Carnitine, Glucose, Caffeine, Guarana, Inositol, Glucuronolactone, Maltodextrin	Caffeine (77 mg)	325 mg Ginko Biloba (150 mg), Caffeine (80 mg), Guarana (25 mg), Inositol (25 mg), L-Carnitine (25 mg), Milk Thistle (20 mg)	Guarana, maltodextrin, caffeine, taurine, panax ginseng, calcium	Citrulline Malate, L-Glutamine, L-Arginine, Quercetin, L-Leucine, L-Valine,

**Table 2:** List of ingredients described on nutrient panels found in some energy shots

**Ingredient**	**A**	**B**	**C**	**D**	**E**
Calories	4	4	20	9	0
Carbohydrate	-	-	4 g	1 g	0
Calories from Fat	-	-	-	-	
Vitamin C	-	100 mg	-	-	126%
Niacin	30 mg	33 mg	33 mg	-	
Vitamin B6	40 mg	40 mg	40 mg	-	
Folic Acid	400 mcg	400 mcg	400 mcg	-	
Vitamin B12	500 mcg	500 mcg	500 mcg	-	
Sodium	18 mg	0 mg	0 mg	-	10
Potassium	-	10 mg	10 mg	-	26
Proprietary	1,870 mg	2,300 mg	2,200 mg	910 mg	527 mg
Blend	Taurine, Glucuronolactone, Malic Acid, N-Acetyl L-Tyrosine, L-Phenylalanine, Caffeine, Citicoline	Glucouronolactone (425 mg), N-Acetyl L-Tyrosine (400 mg), L-Phenylalanine (375 mg), Taurine (350 mg), Malic Acid 300 mg), Caffeine (200 mg), Green Tea Extract (150 mg), Ginseng Extract (150 mg)	Glucouronolactone (420 mg), L-Phenylalanine (380 mg), D-Ribose (350 mg), N-Acetyl L-Tyrosine (325 mg), Malic Acid (300 mg), Caffeine (175 mg), Green Tea Extract (150 mg), Ginseng Extract (100 mg)	Caffeine Citrate, Caffeine Anhydrous, Evoburn, Octapomine, Gugulsterone E & Z, Yerbe Mate, Green Tea, Synephrine, cAMP, Vinpocetine, Yohimbe HCL	Beta-Alanine, Vitamin C, Caffeine Anhydrous (158 mg), Evoburn, N-Acetyl-L-Tyrosine, Hordinine, 5-Hydroxy-L-Trypotophan (5-HTP), Potassium, N-methyl Tyramine, Sulbutlamine, Vinpocetine, Yohimbine HCI, St. John’s Wort Extract

### Caffeine

Caffeine is the most common ingredient utilized in energy drinks. Caffeine is extracted from the raw fruit of over sixty species of coffee plants (*coffea Arabica*), all part of the methylxanthine family. Caffeine is also extracted from tea, kola nuts, and cocoa. After ingestion, caffeine is quickly absorbed and increases in plasma concentrations are generally observed between 30 – 60 minutes following ingestion [7]. The difference in absorption time is dependent on the physicochemical formulation properties of the product dose [8]. Caffeine is a strong cardiovascular stimulant that increases epinephrine output to a greater extent when ingested via its anhydrous formulation when compared to an equal amount of brewed or instant caffeinated coffee [9,10]. In addition, caffeine’s half-life ranges from approximately 2 to 10 hours with 0.5% - 3.5% of its content excreted unchanged in urine and select amounts eliminated via perspiration [11]. A recent position stand from the Journal of the International Society of Sports Nutrition [7] summarized the effects of caffeine on exercise performance as follows:

1. Caffeine is effective for enhancing sport performance in trained athletes when consumed in low-to-moderate dosages (~3-6 mg·kgBM^-1^) and overall does not result in further enhancement in performance when consumed in higher dosages (≥ 9 mg·kgBM^-1^).

2. Caffeine exerts a greater ergogenic effect when consumed in an anhydrous state as compared to coffee.

3. It has been shown that caffeine can enhance vigilance during bouts of extended exhaustive exercise, as well as periods of sustained sleep deprivation.

4. Caffeine is ergogenic for sustained maximal endurance exercise, and has been shown to be highly effective for time-trial performance.

5. Caffeine supplementation is beneficial for high-intensity exercise, including team sports such as soccer and rugby, both of which are categorized by intermittent activity within a period of prolonged duration.

6. The literature is equivocal when considering the effects of caffeine supplementation on strength-power performance, and additional research in this area is warranted.

7. The scientific literature does not support caffeine-induced diuresis during exercise, or any harmful change in fluid balance that would negatively affect performance.

As demonstrated below, several studies have reported significant improvements in both aerobic and resistance exercise with a relative dosage of approximately 2 mg·kgBM^-1^of caffeine. This is less than the amount recommended (3-6 mg·kgBM^-1^) to enhance performance [7], and may contribute to the hypothesis that the synergistic effects of the various ingredients contained in ED/ES are responsible for the reported improvements in exercise performance.

### Carbohydrate

Another common ingredient in most ED is some type of carbohydrate source (e.g., glucose, sucrose, maltodextrin, etc.). Energy drinks also typically contain glucuronolactone, an ingredient which is involved in ascorbic acid synthesis and is metabolized into xylulose [12]. Evidence from numerous studies indicates that carbohydrate feeding during exercise of about 45 minutes or longer can improve endurance capacity and performance [13,14]. Mechanisms by which carbohydrate feeding prior to and during exercise improves endurance performance include maintaining blood glucose levels, maintaining high levels of carbohydrate oxidation, and the sparing of liver and possibly skeletal muscle glycogen [15]. Peak rates of carbohydrate oxidation are commonly around 1 g of carbohydrate per minute or 60 g·hr^-1^. Glucose, sucrose, maltodextrins and amylopectin are oxidized at high rates, while fructose, galactose and amylose are oxidized at lower rates (approximately 25-50% lower) [16]. Consequently, sports drinks typically contain a mixture of various types of carbohydrates designed to optimize exogenous carbohydrate oxidation [17].

ED’s contain approximately 25-30 grams of carbohydrate per 240 mL (8 fluid ounces) serving. This amount nearly meets the lower value of 30 grams/hour recommended during endurance exercise, but falls short of the upper range of 60 g·hr^-1^. In order to meet this upper level of 60 grams of carbohydrate per hour during endurance exercise, approximately 530 mL (18 fluid ounces) of a typical ED per hour would need to be consumed. While the total carbohydrate content of typical ED is quite high, a shortcoming exists in regards to the concentration of commercially available energy drinks. The American College of Sports Medicine [18] and the ISSN [6,17] recommend ingesting carbohydrate in a 6-8% solution (6-8 grams per 100 ml of fluid) during endurance exercise. A typical ED provides carbohydrates at a greater concentration, typically around an 11-12% solution. Ingesting higher percentages (>10%) of carbohydrate in fluids has been reported to delay gastric emptying and increase gastrointestinal distress [19,20]. Consequently, athletes who want to use ED as sports drinks may need to dilute the beverage and/or alternate consumption of ED and water during exercise.

### Other nutrients

Tables 3, 4, and 5 present a list of additional nutrients commonly found in ED or ES. Most ED and ES also contain a small amount of vitamins (e.g., thiamin, riboflavin, niacin, Vitamin B6, Vitamin B12, pantothenic acid, Vitamin C) and electrolytes (e.g., sodium, potassium, phosphorus, etc.). While the addition of these nutrients may add to the nutrient density of these products, there is little evidence that ingestion of these vitamins and minerals in the amounts found in ED and ES would provide any ergogenic benefit during exercise performance in well-nourished individuals [17,18]. Additionally, ED and ES typically contain nutrients purported to promote cognition and mental focus (e.g., Taurine, Ginkgo biloba, L-Tyrosine, Citocoline, 5-Hydroxy-L-Tryptophan [5-HTP], St. John’s Wort, etc.), stimulants (e.g., caffeine, Guarana, Green Tea, Synephrine, Yerba mate, Yohimbine, Tyramine, Vinpocetine, etc.), and/or various purported ergogenic nutrients (e.g., Panax Ginseng, L-Carnitine, D-Ribose, β-Alanine, Inositol, Citrulline, Quercetin, etc.). While there are data to support the potential ergogenic value of some of these nutrients on cognitive function and/or exercise capacity [17,18]; the amounts found in ED and ES are generally much lower than the typical concentrations associated with an ergogenic effect. Consequently, it is unclear whether adding these nutrients to ED and/or ES provides a synergistic or additive effect to the carbohydrate and caffeine found in these products. In addition, adding these nutrients to the caffeine found in ED and/or ES may change the adverse effect profile of these finished products, and warrant further study.

**Table 3:** Potential ergogenic nutrients contained in energy drinks that may affect cognition and/or mental performance

**Ingredient**	**Potential ergogenic value**	**Scientific support**
**Taurine**	Improved mental focus, concentration, serve as antioxidant, glucose homeostasis [21-24]	Some supportive evidence with ED and fed animals [25-35]
**Gingko Biloba**	Improve memory and mental concentration	Some supportive evidence on memory (e.g., 120 mg/d) [36-39]. No known effects at dosages found in ED or ES.
**L-Tyrosine**	Prevents depletion of catecholamines, may ameliorate declines in cognition with acute stress [40-47]	Some supportive evidence on cognition (e.g., 2 g/d, 150 mg acute ingestion with cold exposure) [41,43,46,48,49]. No effects on performance capacity [42,50]. No known effects at dosages found in ED or ES.
**Citicoline**	Intermediate in the generation of phosphatidylcholine from choline. Increase dopamine receptor densities and delay memory impairment [51,52].	Some supportive evidence with large doses (8.5 g prior to and during exercise) and in fed animals [52]. No known effects at dosages found in ED or ES.
**5-Hydroxy-L-Trypotophan (5-HTP)**	Precursor to serotonin [53,54]. Purported antidepressant, appetite suppressant, & sleep aid [53,55-58].	Some evidence in treatment of depression [53,55-58]and 5-HT fed animals on muscle performance [54,59,60]. Role on exercise performance at dosages found in ED and ES is unknown.
**St. John’s Wort**	Anti-depressant [56-58].	Some supportive evidence [56-58]. No known effects at dosages found in ED or ES.

**Table 4:** Potential stimulants contained in energy drinks that may affect performance capacity

**Ingredient**	**Potential ergogenic value**	**Scientific support**
**Caffeine**	Stimulant. Increases metabolism and lipolysis [2,8,9,61].	Increases alertness, mood, cognitive function [2,8,9,61]. Increases fat oxidation, spares glycogen utilization, improves exercise [7,9-11,62-65].
**Guarana**	Natural source of caffeine. Similar properties to caffeine.	Similar to caffeine effects.
**Green Tea Extract**	Contains high amounts of caffeine and catechin polyphenols (e.g., epigallocatechin gallate or EGCG). Serves as antioxidant. Similar effects as caffeine [66,67]	Some supportive evidence of increased metabolism [68-76]. Specific role at dosages found in ED is unknown.
**Synephrine**	Alternative to ephedrine. Naturally derived from *Citrus aurantium*. Stimulant with less cardiovascular effects than ephedrine. Purported to increase metabolism and promote weight loss.	Evidence of a mild stimulant effect on metabolism and weight loss [77-82]. No known effects at dosages found in ED.
**Yerba mate**	Contains three xanthines (caffeine, theobromine, and theophylline). Similar properties to caffeine	Similar to caffeine effects. Some supportive evidence [83-85] No known effects at dosages found in ED and ES.
**Yohimbine**	Alkaloid with stimulant and aphrodisiac properties [86-90].	Similar to caffeine effects. Effects at dosages found in ED are unknown.
**Tyramine**	Naturally-occurring monoamine derived from tyrosine. Acts as a catecholamine (dopamine, NE, Epi) releasing agent. Degraded to octopine. Increases blood pressure and can serve as neurotransmitter [91-93].	Mild cardiovascular stimulant. Effects at dosages found in ED / ES are unknown.
**Vinpocetine**	Alkaloid of vincamine extracted from periwinkle plant (Vinca) minor. Vasodilatory and memory enhancing properties [94,95].	No known effects at dosages found in ED or ES.

**Table 5:** Other potential ergogenic nutrients contained in energy drinks that may affect performance

**Ingredient**	**Potential ergogenic value**	**Scientific support**
**Panax Ginseng**	Contains ginsenosides which are purported to have anti-inflammatory, antioxidant, and anticancer effects. Purported to enhance perceptions of energy, increase stamina and improve nitrogen balance [96].	Most well-controlled research does not support the ergogenic effects for ginseng [97-111]. No known effects at dosages found in ED and ES.
**L-Carnitine**	Involved in shuttling long chain fatty acids into mitochondria. Purported to promote lipolysis [112].	Limited supportive ergogenic value in athletes or on weight loss [112]. No known effects at dosages found in ED and ES.
**D-Ribose**	Involved in ATP synthesis. Theoretically, D-ribose supplementation can increase ATP availability.	Some evidence of improved exercise capacity in clinical populations [113] but limited evidence that high dose ribose supplementation affects exercise capacity [114-119]. No known effects at dosages found in ED and ES.
**Beta Alanine**	Increases muscle carnosine levels, increases muscle buffering, and attenuates fatigue during high intensity exercise [120-124].	Growing scientific evidence of improved anaerobic capacity (2-4 g/d) [125-138]. No known effects at dosages found in ED and ES.
**Inositol**	Carbohydrate that is not classified as sugar. Involved in insulin signaling, nerve transmission, serotonin modulation, and fat oxidation [139].	No known effects at dosages found in ED or ES.
**Citrulline Malate**	Optimizes blood flow via arginine-nitric oxide pathway; purported to reduce fatigue and buffer acidity during exercise [140,141].	Some evidence that high dosages (e.g., 6 – 8 g) can affect exercise capacity and/or anabolism [142-149]. No known effects at dosages found in ED and ES.
**Quercetin**	Reported to have antioxidant, anti-inflammatory, antiviral, and immune-modulatory effects [150].	Several studies indicate that Quercetin supplementation (e.g., 1 g/d for 7 d) increases maximal aerobic capacity and time to fatigue [151-166]. No known effects at dosages found in ED or ES.

## Exercise performance

Several studies have investigated the effects of ED consumption prior to exercise. The types of exercise that were evaluated include resistance exercise [167,168], anaerobic exercise [169], and aerobic/endurance exercise [62,170-172].

### Ingestion prior to anaerobic exercise

Many of the studies investigating the effects of ED ingestion on anaerobic performance measures have been conducted within the past several years. In a crossover study (separated by seven days), Forbes and colleagues [168] gave 15 physically active college-aged students a commercially available energy drink standardized with 2 mg·kgBM^-1^of caffeine or an isoenergetic, isovolumetric, non-caffeinated placebo 60-minutes prior to exercise. The exercise consisted of three sets of 70% one repetition maximum (1RM) bench press conducted to failure on each set with one minute of rest between each set. Following the resistance exercise bout, three x 30-second Wingate Anaerobic Capacity tests were also conducted with two minutes of rest between each test. The ED significantly increased total bench press repetitions over three sets (approximately 6% more repetitions completed) but had no effect on Wingate peak or average power.

In a similarly designed study, a commercially available energy drink (providing an average of 2.1 mg of caffeine per kg of body mass) given to physically active male and female participants 45 minutes prior to exercise resulted in a significant increase in leg press total lifting volume (12% increase as compared to a carbohydrate placebo) but had no effect on bench press total lifting volume [167] or multiple 20-second Wingate-type cycle sprints [173]. Hoffman and colleagues [169] gave male strength/power athletes an ED containing an average of 1.8 mg·kgBM^-1^of caffeine or a placebo beverage that was similar in taste and appearance but contained only inert substances. Following the ingestion of the ED, three separate 20-second Wingate tests separated by about 15 minutes were performed. Results revealed that there were no significant differences between trials in any anaerobic power measure. In a recent publication, 12 healthy male and female non-resistance trained participants ingested a commercially available ED standardized at either 1 or 3 mg·kgBM^-1^of caffeine or a placebo beverage (containing no caffeine) in a randomized, repeated measures design [65]. Sixty minutes following beverage ingestion, each participant completed 10-to-100% 1RM power-load tests for the bench press and half-squat. Ingestion of the ED with 1 mg·kgBM^-1^of caffeine was not enough to raise the power output during the power-load tests. However, the ingestion of an ED with 3 mg·kgBM^-1^of caffeine increased maximal power output by 7% in both the half-squat and bench-press as compared to the ingestion of a placebo [65]. A recent study by Gonzalez and colleagues [174] indicated that an energy matrix consisting of caffeine, taurine and glucoronolactone consumed 10-min prior to a workout resulted in an 11.9% improvement (p < 0.05) in the number of repetitions performed during 4 sets of the squat or bench press exercise using 80% of the subject’s 1-RM. In addition, the average power output for the workout was significantly higher for subjects consuming the energy drink compared to subjects consuming the placebo.

In addition to resistance and high intensity anaerobic exercise, the effects that ED exert on speed/agility performance has also been investigated. Collegiate female soccer players ingested an ED containing 1.3 mg·kgBM^-1^of caffeine and 1 gram of taurine or a caffeine and taurine-free placebo 60 minutes prior to repeated agility t-tests [175]. No difference in agility *t*-test performance between the ED and placebo groups was reported. Specifically, the highest difference reported between the two groups was during the third set of eight agility t-tests, and the difference reached only 1.15% between the groups. It is unlikely that the carbohydrate content alone in ED is responsible for improvements in resistance exercise performance. In support of this view, the majority of studies in which supplemental carbohydrate was ingested prior to a resistance-training bout did not report improvements in resistance training performance [176-178].

### Conclusion

ED (containing approximately 2 mg·kgBM^-1^caffeine) consumed 45 to 60 minutes prior to anaerobic/resistance exercise may improve upper- and lower- body total lifting volume, but has no effect on repeated high intensity sprint exercise, or on agility performance.

### Ingestion prior to endurance exercise

Several studies have investigated the effects of ED ingestion prior to aerobic exercise [62,170-172,179]. In the earliest of these studies, Alford and colleagues [172] investigated the effects of ingesting a commercial ED on aerobic endurance. In a repeated measures, crossover design, young healthy participants ingested 250 mL of a commercial ED (containing 80 mg of caffeine and 26 grams of carbohydrate), a carbonated water beverage, or no beverage at all 30 minutes prior to performing an endurance exercise bout. Test days for separate treatments were assessed within a week. Aerobic performance was analyzed by the amount of time that exercise could be maintained at 65-75% of maximum heart rate on a cycle ergometer. Significant improvements in aerobic performance were reported for the commercial ED treatment. Aerobic performance was 8% and 14% longer after ingesting the commercial ED as compared to the carbonated water and no beverage treatment, respectively.

In one of only two studies that have investigated the effects of ingesting a sugar/carbohydrate-free ED on performance capacity, Candow and colleagues [170] reported no improvements in high intensity run time-to-exhaustion performed at 80% of VO_2_max on a treadmill in physically active college-aged participants. The sugar-free ED contained 2 mg·kgBM^-1^caffeine and was ingested one-hour prior to the exercise bout [170]. In contrast, Walsh and colleagues [179] reported significant improvements in treadmill run time to exhaustion following ingestion of a carbohydrate-free ED. In this randomized cross-over investigation, 15 recreationally active participants ingested an ED 10-minutes prior to engaging in a treadmill run-to exhaustion test at 70% VO_2_max [179]. The ED utilized in this study did not contain any carbohydrate, and unlike other ED products, contained nearly eight grams of the amino acids L-leucine, L-isoleucine, L-valine, L-arginine and L-glutamine. Unfortunately, the published study did not disclose the precise amount of caffeine contained in the ED, but instead referred to a ~2 g “proprietary blend” of caffeine, taurine, and glucoronolactone. The placebo used as a comparison was sweetened water that was similar in color and volume. It was reported that participants consuming the ED were able to run 12.5% longer than during the placebo treatment [179].

The two most common protocols used to assess aerobic performance are time to exhaustion at a given exercise intensity (e.g., exercise at 70% of maximum oxygen uptake until exhaustion) and time trial performance for a set distance (e.g., 40 km time trial). Time trials have greater validity than time to exhaustion because they provide a good physiological simulation of actual performance and correlate with actual performance [180,181]. Ivy and colleagues [62] were the first research group to utilize a time trial component in conjunction with ED consumption. In this investigation, trained male and female cyclists completed two trials in a repeated measures crossover design separated by one week. After a 12 hour fast, the cyclists ingested a commercially available ED providing approximately 2.3 mg·kgBM^-1^caffeine or an artificially colored, flavored, and sweetened-water placebo 40-minute prior to the exercise bout. Performance during the exercise bout was measured as the time to complete a standardized amount of work equal to 1 hr of cycling at 70% of maximal power output. Results revealed a significant difference between the treatments in relation to performance with the ED treatment completing the time trial ~4.7% faster than the placebo treatment [62].

### Conclusion

ED containing approximately 2 mg·kgBM^-1^caffeine consumed 10 to 40 minutes prior to aerobic exercise improve cycling and running performance in both trained cyclists and recreationally active participants. In the one investigation in which no aerobic performance improvement was reported, the ED (containing 2 mg·kgBM^-1^caffeine) was ingested 60-minutes prior to the performance assessment. In light of the other findings, ingestion of the caffeine-containing ED 60-minutes prior to the exercise bout may be too long of a period to realize improvements in aerobic exercise performance.

### Mood/reaction time/alertness

Reaction time, concentration, alertness, and subjective feelings of energy/vitality are important in many competitive activities such as hitting a baseball, returning a serve in tennis, and dodging strikes and kicks in a mixed martial arts competition. Strategies to improve these attributes are often sought after by individuals competing in certain athletic endeavors. Over the past several years, research has investigated the effects that ED ingestion has on these (and other) variables.

Seidl and coworkers [31] conducted a study utilizing three common ingredients (i.e., caffeine, taurine, glucuronolactone) typically found in ED and compared it to a placebo group. Participants were evaluated at night to see if ingestion of these nutrients affected mood and motor function in fatigued participants. Interestingly, the investigators found that at the end of the experiment, reaction time was significantly longer in the placebo group, but remained unchanged in the group that consumed the ED ingredients. Similarly, vitality scores, feelings of well-being, and social extrovertedness were all significantly decreased in the placebo group, but did not change in the ED group [31].

Scholey and colleagues [182] investigated the effects of an ED (containing primarily caffeine, glucose, ginseng and ginkgo biloba drink) or a placebo beverage on five aspects of cognitive performance and mood. Thirty minutes after consuming ED, two of the five variables (i.e., “secondary memory” and “speed of attention”) were significantly improved as compared to the placebo beverage [182]. Other investigators also reported that when caffeine was combined with carbohydrates in a carbonated beverage, performance and mood were improved and/or maintained during fatiguing and cognitively demanding tasks relative to placebo [183]. Similarly, ED containing caffeine and glucose have also been shown to enhance event related potentials (i.e., a measure of brain activity in real time obtained from an electroencephalogram), which may translate to improvements in reaction time [184].

Hoffman and colleagues [169] reported that when male strength/power athletes consumed 120 ml of a commercially available ED or a placebo, reaction time and subjective feelings of energy and focus were significantly improved in those consuming the ED. Furthermore, the investigators also noted a statistical trend (*p=0.06*) towards an increase in alertness. In a similar study, Walsh and colleagues [179] examined the effects of ingesting an “energy matrix” (2.05 g of caffeine, taurine, glucuronolactone), amino acids (7.9 g of L-leucine, L-isoleucine, L-valine, L-arginine and L-glutamine), di-creatine citrate (5 g), and β-alanine (2.5 g) mixed with 500 ml of water or a placebo) 10-minutes prior to exercise on aerobic performance and subjective measures of focus, energy, and fatigue in recreationally active male and females. Results revealed that participants ingesting the ED increased time to exhaustion while running at 70% of VO_2_max by 12.5% (p = 0.012), they reported greater focus (p = 0.031), energy (p = 0.016), and less fatigue (p = 0.005) prior to exercise; and, that their ratings of focus (p = 0.026) and energy (p = 0.004) were greater 10 minutes into exercise [179]. However, no significant differences in energy, fatigue, and focus were observed between groups immediately post-exercise [179].

Howard and coworkers [185] evaluated the effects of acute ingestion of a glucose containing ED on behavioral control. In this study, 80 participants were randomly assigned to consume 1.8, 3.6, or 5.4 ml/kg of an ED, a placebo, or no drink in a counterbalanced manner. Participants completed a behavioral control task and subjective measures of stimulation, sedation, and mental fatigue before and 30-minutes after ingestion of the assigned drinks. Results revealed that those consuming the ED decreased reaction times on the behavioral control task, increased subjective ratings of stimulation and decreased ratings of mental fatigue. The greatest improvements in reaction times and subjective measures were observed with the lower dose and improvements diminished as the dose increased. Earlier research conducted by Alford and associates [172] supported these findings by demonstrating that individuals ingesting 250 ml of this same ED had significantly better reaction time, concentration, memory, and subjective alertness compared to a placebo. Smit and coworkers [183] suggested that caffeine is most likely the primary ingredient that improves mood and performance during fatiguing and cognitively demanding tasks, with carbohydrates playing a minor role. However, caffeine and carbohydrate may act in a synergistic manner [182]. To support this view, a recent paper by Pettitt et al [186] reported that while ingestion of an ED prior to exercise affected aerobic metabolism during and following cycling exercise, the secondary ingredients found in the ED had no additive effects.

### Conclusion

To date, most studies on ED have reported improvements in mood, reaction time, and/or markers of alertness, even though the relative importance of the various ingredients is not fully understood. The primary ergogenic value appears to be due to the caffeine and/or carbohydrate contained in these drinks. Individuals looking to enhance reaction time, mental alertness, and/or focus may benefit from consuming an ED prior to exercise.

## Energy drinks and their role in energy expenditure and weight loss

As shown in Table 4, ED and some commercial beverages designed to increase metabolism typically contain a number of stimulants (e.g., caffeine, Guarana, Green Tea, synephrine, Yerba mate, Yohimbine, Tyramine, Vinocetine, etc.). Several low-calorie ED and beverages have been marketed as “thermogenic blends” with a focus on increasing metabolism. Theoretically, ingestion of ED prior to exercise may increase energy expenditure which over time could help manage and/or promote weight loss. In support of this theory, studies have shown that ingestion of caffeine (e.g., 200-500 mg) can increase acute (1-24 hours) energy expenditure [187-193], chronic (28 days) energy expenditure [194], and elevate plasma free-fatty acid and glycerol levels [187,194,195]. Collectively, these findings suggest that the stimulant properties of caffeine contained in ED can elevate an individual’s metabolic rate as well as elevate the rate of lipolysis in the body. However, these studies used various types of caffeine/stimulant/vitamin-enriched coffee [189-193], a caffeine/stimulant blend supplement [187,189,193], and various calorie-free thermogenic ED [190,194-197]. Additionally, the dosage of caffeine used in some of these beverages that are marketed as a thermogenic supplements is typically higher (e.g., 200-500 mg) than the concentrations found in ED and ES marketed for increasing athletic performance or alertness (i.e., about 80 – 200 mg). With this said, there is some data that indicates that acute ingestion of ED has been shown to enhance energy expenditure, metabolic rate, catecholamine secretion, and/or lipolysis [187,198]

In terms of weight loss, Roberts and colleagues [194] reported that 28 days of consumption of a calorie free ED (336 ml/day) promoted small (i.e., 18.9 ± 1.5 to 18.3 ± 1.5 kg) but statistically significant (p<0.05) reductions in fat mass compared to controls (i.e., 18.1 ± 1.3 to 18.4 3± 1.2 kg). Similarly, Stout and associates [199] evaluated the effects of consuming an ED or placebo 15-minutes prior to exercise training and ad-libitum on non-training days for 10-weeks on changes in body composition and fitness. Results revealed that those consuming the ED experienced greater changes in fat mass (-6.6% vs. -0.35%, p<0.05), peak aerobic capacity (+13.8% vs. 5.4%, p<0.01), and treadmill time to exhaustion (+19.7% vs. 14.0%, p<0.01). These findings suggest that consumption of ED during training and/or weight loss may provide some additive ergogenic benefits. However, it should be noted that recent review on ED by Higgins and associates [200] found that many of the commonly used additional ingredients (e.g., Ma Huang, willow bark, synephrine, calcium, cayenne/black pepper extracts) that are contained in the “thermogenic blends” of several of these products are not contained in some of the most commonly used ED. It is also important to note that daily consumption of high calorie ED could promote weight gain. Consequently, additional research is necessary to determine whether ingesting low-calorie ED or ES may affect training adaptations and/or weight loss.

### Conclusion

Consumption of low calorie ED and thermogenic beverages have been reported to increase resting energy expenditure and fat metabolism on an acute basis. Preliminary studies suggest that ingesting some types of ED and thermogenic beverages prior to exercise during training could promote positive adaptations in body composition. However, more research is needed to determine whether daily use of ED would affect long-term energy balance and body composition.

## Safety considerations

ED have had a negative connotation in the media and more recently medical community, mostly related to potential concerns about excessive caffeine intake [201,202] and/or potential deleterious effects of mixing ED with alcohol [203]. While safety concerns and use of alcohol go beyond the scope of this paper, the reader is referred to a recent viewpoint published in the Journal of the American Medical Association related to safety concerns of mixing ED with alcohol [203]. In terms of use of ED in the traditional sense, most concerns have been based on case studies or adverse event reports that have serve only to document a potential association, but does not establish causality. In reality, there are currently only a few studies (acute or long term) that have investigated the side effects of ED [204-209]. There appear to be two primary active nutrients in most ED and ES (i.e., carbohydrate and caffeine) that may possess safety concerns in some populations. Many ED contain 25 – 50 g of simple sugars, therefore, ingestion of ED prior to exercise are likely to rapidly increase insulin in order to maintain normal blood glucose levels. For this reason, diabetics and pre-diabetics should avoid high glycemic load ED or consider consuming low carbohydrate versions of ED [201,202].

Very often, ED also contain various stimulants with the most common being caffeine. Some concern has been raised about excessive caffeine intake that could be obtained from consuming too many ED and/or from a lack of knowledge that that some ingredients contained in ED may contain caffeine [201,202]. Currently in the United States, the FDA has regulated the limit of caffeine in soft drinks to 0.02 percent (10mg/oz.) of the product, but this is not currently enforced for ED or ES. As of December 2012, the US-FDA along with the US Congress has begun to study products marketed as ED or ES, however no formal new guidelines have been published. The Nutrition Facts Panel on food labels are not required to always list caffeine since it is not a nutrient. However, if caffeine is added to a food, it must then be listed [210]; therefore many individuals may consume more caffeine than they realize [201,202]. In Canada, caffeine levels are limited to 180 mg per drink [211]. The caffeine content of common ED and ES has been reported to range from about 100 to 286 mg [202]. As a comparison, the average cup of coffee or contains between 40 and 150 mg caffeine, while a 20 oz. cup of Starbucks regular drip coffee has been found to contain as much as 480 mg of caffeine [212].

The potential side effects of caffeine include: insomnia, nervousness, restlessness, gastric irritation, nausea, vomiting, tachycardia, tremors, and anxiety; which have been reported at doses as low as 250 to 300 mg [5,201-204,209]. Caffeine availability is ubiquitous and it is one of the most extensively studied substances in the food supply with a long history as generally regarded as safe when consumed in moderation [61]. However, all substances may be toxic under the right conditions, with toxicity being a function of the interaction of many physiologic variables that include the following: acute and chronic dosing, route of administration, genetics, age, sex, environment, and intrinsic health of the individual being exposed. Young adults have been found to have subclinical coronary atherosclerosis [213]. In addition, post-mortem assessment of sudden cardiac death in young persons (<35 years) reveals a variety of anatomic abnormalities of the coronary arteries, myocardium, valves and the conduction system [214]. Such unknown pre-existing risk factors may increase the risk of adverse events, particularly cardiovascular ones, in individuals consuming EDs, due to underlying disease. In fact, even water can be toxic given certain conditions with an LD_50_ (lethal acute dose for 50 percent in test species) of greater than 90 mL/kg in rats [215]. It is possible to overdose on caffeine and there are a handful of case reports in the literature [5,209,216-218]. A lethal dose of caffeine has been typically in excess of 5 g [217], which equates to about 42 cups of coffee at 120 mg of caffeine per cup. Sepkowitz [201] recently suggested that an intake of 3 grams of caffeine (equivalent to ingesting 12 or so highly caffeinated ED within a few hours) could elicit significant adverse effects. The average caffeine per serving in most ED and ES range between 75 and 200 mg, an amount similar to the caffeine found in a premium cup of coffee [202].

Nawrot and colleagues [219] stated that in a healthy adult population, up to 400 mg of caffeine daily was not associated with any adverse effects. In another review, Higdon et al. [220] presented data in children stating no adverse effects were seen with doses under 3 mg·kgBM^-1^·day^-1^. As with most drugs, the exact amount of caffeine where side effects will occur varies from person to person based on genetics, age, liver cytochrome P450-CYP1A2 isozyme function, concurrent medications or substances that may affect hepatic metabolism, body mass, and sensitivity. Additionally, it is unknown whether inclusion of other stimulants in ED and/or ES may increase or decrease the threshold for experiencing side effects. For this reason, some groups do not recommend ED or ES for athletes participating in exercise lasting less than 1 hour [200], despite the admission of inadequate long-term data. The longest duration studies on ED or ES we were able to find was 10 weeks and these studies did not report any change in clinical safety markers [199,206]. Nevertheless, since ED and ES often contain other stimulants that can have a synergistic effect with caffeine, more research is needed to determine the long-term effects of habitual intake of ED and ES before definitive conclusions can be drawn.

Several reports have expressed concern about the safety of ED [5,200,205,221]. For example, Worthley and associates [222] tested 50 young male and female adults one hour before and one hour after consuming 250 ml of a sugar-free ED containing approximately 80 mg of caffeine. The investigators found that mean arterial pressure increased by approximately 3.8 mmHg while resting heart rate was not affected. Additionally, platelet aggregation increased by 13.7% compared to only a 0.3% change in the control group while endothelial function decreased. The researchers noted that the component of the ED that was associated with these results was not clear. However, they suggested that since endothelial dysfunction and impaired platelet function are associated with elevated glucose levels, it is possible that glucuronolactone contained in the ED might have contributed to the observed detrimental effects of energy drinks [222]. More research is needed to corroborate these findings as well as to determine whether these acute changes would pose any long-term health risk.

Bichler and cohorts [26] investigated a combination of caffeine and taurine (two common ingredients in ED) in a double-blind study of college students. Subjects consumed either caffeine and taurine pills or a placebo and then completed a memory assessment while heart rate and blood pressure were monitored. The combination caused a significant **decline** in heart rate and an increase in mean arterial blood pressure. Steinke et al. [223] studied 15 healthy adults who abstained from caffeine for 48 hours prior to and during the study in addition to being fasted overnight. Baseline measurements of blood pressure and heart rate were measured. On day one of the study, each participant consumed 500 mL (2 cans) of an ED and measurements were repeated 30 minutes, 1 hour, 2 hours, 3 hours, and 4 hours later. Participants also drank 500 mL of the ED drink daily for the next 5 days. The experiment was then repeated after 7-days. The investigators found that maximum mean heart rate occurred at 4 hours with significant increases of 7.8% and 11.0% on days 1 and 7, respectively. Blood pressures were increased approximately 7% after acute ingestion of the ED on day 1 (significant increase) but no differences were seen on day 7. Finally, in a case report, Usman and coworkers [221] reported that a young boy presented with palpitations and high blood pressure after consumption of an ED containing carbohydrate (40 g), sodium citrate, taurine (124 mg), caffeine, inositol (17 mg), Panax ginseng (6.98 mg), and other nutrients. The tachycardia and hypertension returned to normal after discontinuation of ED consumption.

### Conclusion

Individuals with certain medical conditions (e.g., metabolic syndrome or diabetes mellitus) should avoid consumption of high glycemic drinks and/or foods and therefore should not consume the high calorie versions of ED. It would be prudent for individuals with known cardiovascular disease to avoid altogether their use of ED and/or ES, or other products with known cardio-stimulant effects. While ED containing caffeine and other stimulants may have negative effects upon health and cardiac parameters in individuals with such pre-existing health conditions, the current evidence (although small) suggests that consumption of ED and ES are safe in healthy populations and similar to ingesting other foods and beverages containing caffeine. Finally, although it is estimated that only 1% of all dietary supplement adverse events are reported to FDA [224], given the number of servings of these products that are consumed daily, the rate of adverse events appears low in the population of consumers. Nevertheless, it is acknowledged that additional short- and long-term studies are needed to better determine any factors that increase the risk for adverse events. Additionally, since ED often contain several nutrients that contain caffeine and/or other stimulants, care should be taken to make sure that an excessive number of ED are not consumed within a short period of time.

## Conclusions and recommendations

Based on a review of the available scientific and medical literature related to the safety and efficacy of the use of ED or ES, the Research Committee of the Society makes the following conclusions and recommendations.

1. Although ED and ES contain a number of nutrients that are purported to affect mental and/or physical performance, the primary ergogenic nutrients in most ED and ES appear to be carbohydrate and/or caffeine.

2. The ergogenic value of caffeine on mental and physical performance has been well-established but the potential additive benefits of other nutrients contained in ED and ES remains to be determined.

3. Consuming ED 10-60 minutes before exercise can improve mental focus, alertness, anaerobic performance, and/or endurance performance.

4. Many ED and ES contain numerous ingredients; these products in particular merit further study to demonstrate their safety and potential effects on physical and mental performance.

5. There is some limited evidence that consumption of low-calorie ED during training and/or weight loss trials may provide ergogenic benefit and/or promote a small amount of additional fat loss. However, ingestion of higher calorie ED may promote weight gain if the energy intake from consumption of ED is not carefully considered as part of the total daily energy intake.

6. Athletes should consider the impact of ingesting high glycemic load carbohydrates on metabolic health, blood glucose and insulin levels, as well as the effects of caffeine and other stimulants on motor skill performance.

7. Children and adolescents should only consider use of ED or ES with parental approval after consideration of the amount of carbohydrate, caffeine, and other nutrients contained in the ED or ES and a thorough understanding of the potential side effects.

8. Indiscriminant use of ED or ES, especially if more than one serving per day is consumed, may lead to adverse events and harmful side effects.

9. Diabetics and individuals with pre-existing cardiovascular, metabolic, hepatorenal, and neurologic disease who are taking medications that may be affected by high glycemic load foods, caffeine, and/or other stimulants should avoid use of ED and/or ES unless approved by their physician.

## Competing interests

BC has received university and private sector funded grants to conduct research on several dietary supplements and has received compensation for speaking at conferences and writing lay articles/books about dietary supplements. PLB has received compensation for contributing to edited books in relation to sports nutrition. CW has received academic and industry funding related to dietary supplements and honoraria from speaking engagements on the topic. LT has received academic and industry funding related to dietary supplements and honoraria for speaking at conferences. MTN declares no competing interests. MG has received academic and industry funding related to dietary supplementation but declares no competing interests regarding the contents of this manuscript. TNZ has received funding from the dietary supplement industry to conduct clinical research through The Center for Applied Health Sciences, has consulted for several dietary supplement companies, and currently serves as a scientific advisor to Biotest Laboratories. HLL has received funding from industry to conduct clinical research through The Center for Applied Health Sciences, has consulted for multiple dietary supplement and medical food companies, and currently serves as scientific and medical advisor to Nordic Naturals, Inc. JRS serves as a science advisor for Abbott Nutrition. SS has not competing interest to declare. RC has no competing interests to declare. DSK works for a Contract Research Organization that receives funding for clinical trials from the pharmaceutical and nutritional industries, serves as a Nutrition Consultant currently to the United States Tennis Association (USTA), Boca Raton, Florida, and serves as the also as the Florida International University, Department of Athletics, Sports Nutritionist. JA is a Sports Science Advisor to VPX/Redline in Weston FL. RBK has received external funding from industry through the institutions he has been affiliated with to conduct exercise and nutrition research, has served as a legal expert on exercise and nutrition related cases, and currently serves as a scientific advisor for Woodbolt International.

## Authors’ contributions

RBK prepared and delivered the presentation on energy drinks at the 2011 International Society of Sports Nutrition (ISSN) National meeting. BC, CW, LT, MTN, and MG developed the presentation into a draft of a position stand for review and editing by RBK. The final draft was then reviewed and edited by TZ, HL, JRH, JRS, SS, RC, DSK and JA. RBK incorporated recommendations into a final draft which was then reviewed, approved, and adopted as the official position of the ISSN by the Research Committee. All authors read and approved the final manuscript.
